# The Molecular Mechanisms Responsible for Tear Hyperosmolarity-Induced Pathological Changes in the Eyes of Dry Eye Disease Patients

**DOI:** 10.3390/cells12232755

**Published:** 2023-12-01

**Authors:** Carl Randall Harrell, Lisa Feulner, Valentin Djonov, Dragica Pavlovic, Vladislav Volarevic

**Affiliations:** 1 Regenerative Processing Plant, LLC, 34176 US Highway 19 N, Palm Harbor, FL 34684, USA; dr.harrell@regenerativeplant.org; 2 Advanced Eye Care & Aesthetics, 104 Plumtree Rd Suite 107, Bel Air, MD 21015, USA; lisafeulner@yahoo.com; 3 Institute of Anatomy, University of Bern, Baltzerstrasse 2, 3012 Bern, Switzerland; valentin.djonov@unibe.ch; 4 Departments of Genetics and Microbiology and Immunology, Center for Harm Reduction of Biological and Chemical Hazards, Faculty of Medical Sciences, University of Kragujevac, 69 Svetozar Markovic Street, 34000 Kragujevac, Serbia; dragica.miloradovic8@gmail.com

**Keywords:** tear hyperosmolarity, dry eye disease, corneal epithelial cells, eye inflammation, hypo-osmotic eye drops

## Abstract

Dry eye disease (DED) is a multifactorial disorder of the lacrimal system and ocular surface, characterized by a deficiency in the quality and/or quantity of the tear fluid. The multifactorial nature of DED encompasses a number of interconnected underlying pathologies, including loss of homeostasis, instability and hyperosmolarity of the tears, and the induction and propagation of detrimental inflammatory responses in the eyes, which finally results in the development of neurosensory dysfunction and visual disruption. Dryness, grittiness, scratchiness, discomfort, inflammation, burning, watering, ocular fatigue, pain, and decreased functional visual acuity are common symptoms of DED. Eye dysfunction drastically attenuates patients’ quality of life. Accordingly, a better understanding of the pathogenic processes that regulate the development and progression of DED is crucially important for the establishment of new and more effective DED-related treatment approaches, which would significantly improve the quality of life of DED patients. Since the process of osmoregulation, which guards the ocular surface epithelia and maintains normal vision, is affected when the osmolarity of the tears is greater than that of the epithelial cells, tear hyperosmolarity (THO) is considered an initial, important step in the development, progression, and aggravation of DED. In order to delineate the role of THO in the pathogenesis of DED, in this review article, we summarize current knowledge related to the molecular mechanisms responsible for the development of THO-induced pathological changes in the eyes of DED patients, and we briefly discuss the therapeutic potential of hypo-osmotic eye drops in DED treatment.

## 1. Introduction

Dry eye disease (DED), also known as keratoconjunctivitis sicca or dysfunctional tear syndrome, is a widespread, multifactorial disorder of the lacrimal system and ocular surface, characterized by a deficiency in the quality and/or quantity of the tear fluid [1].

The ocular surface is highly exposed to environmental threats, which are efficiently eliminated by the tears [2]. Accordingly, an efficient generation and adequate turnover of tears is crucially important for proper eye function [2]. The lacrimal functional unit (LFU) is composed of the lacrimal and meibomian glands, mucin-producing goblet cells, ocular surface secretory cells, lacrimal outflow pathways, and corneal and conjunctival epithelial cells [3]. All components of the LFU work together to maintain the tear film, protect the transparency of the cornea, and preserve the integrity of the ocular surface [3]. It is important to note that the LFU interacts with the neurological and endocrine systems and is not a standalone system. Destabilizing the tear film will result in the development of DED if any LFU component is damaged, as well as if neural and endocrine disorders (such as sensory and motor nerve dysfunction, or hormonal imbalance) arise [3]. An unbalanced tear film’s inability to appropriately nourish or protect the ocular surface results in permanent damage to the corneal and conjunctival epithelial cells and corneal nerve fibers [1,3].

There are two main subtypes of DED: evaporative dry eye (EDE), which is typically attributable to excessive evaporation of the tear fluid; and aqueous tear-deficient dry eye (ADDE), which is characterized by inefficiency or failure of the lacrimal glands to generate tears [4]. While the cause of ADDE may be autoimmune or related to a breach in the integrity of the LFU, EDE usually develops due to meibomian gland dysfunction (MGD) [4]. In clinical settings, DED is frequently observed as a “hybrid” or “mixed” version of these two subtypes, wherein each subtype acquires some of the clinical traits of the other, initiating and aggravating its pathology [4].

**Figure 1:**
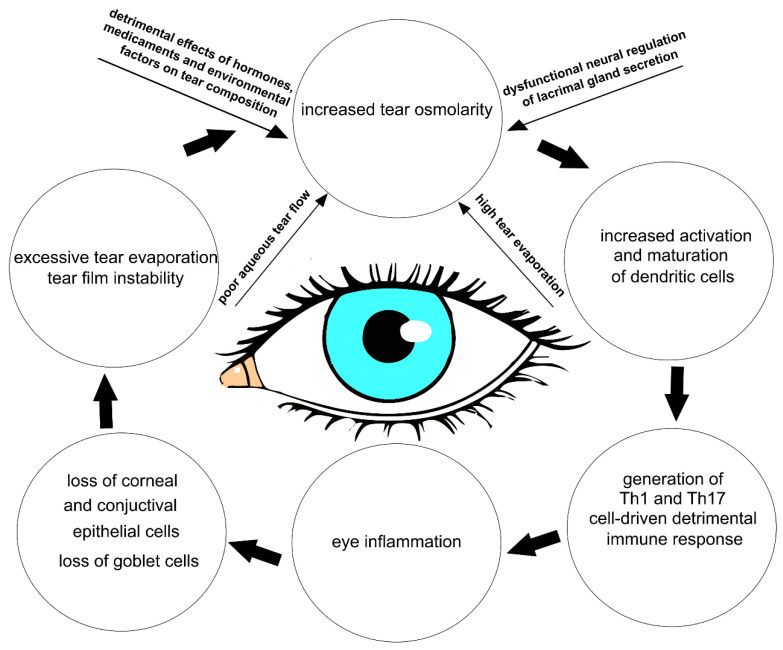
Crucial factors contributing to the development of THO and DED. Hormones, medicaments, environmental factors, dysfunctional neural regulation of lacrimal gland secretion, poor aqueous tear flow, and excessive tear evaporation result in the development of THO. THO induces increased activation and maturation of dendritic cells, which generates a detrimental Th1 and Th17 cell-driven immune response in the eyes of DED patients. Through the production of inflammatory cytokines, Th1 and Th17 cells induce inflammation, which causes injury to the epithelial and goblet cells in the eyes of DED patients. The loss of goblet, corneal, and conjunctival epithelial cells results in excessive tear evaporation and tear film instability, which crucially contribute to the development of THO and DED.

The multifactorial nature of DED encompasses a number of interconnected underlying pathologies, including a loss of homeostasis, instability and hyperosmolarity of the tears, and the induction and propagation of detrimental inflammatory responses in the eyes, which finally results in the development of neurosensory dysfunction and visual disruption [1,5]. These detrimental events create a “pathological loop” that promotes the development and exacerbation of DED (Figure 1) [1].

Dryness, grittiness, scratchiness, discomfort, inflammation, burning, watering, a feeling of a foreign substance in the eye, ocular fatigue, pain, and decreased functional visual acuity are all common symptoms of DED [5,6]. Eye dysfunction drastically attenuates patients’ quality of life and impairs patients’ abilities to do daily tasks that depend on their vision.

Long-term symptom relief is currently the main therapeutic goal of DED treatment, along with reestablishing the natural equilibrium on the surface of the eye [7]. Accordingly, tear replacement medications are increasingly offered to DED patients in order to attenuate dry eye-related symptoms [8]. However, the complex etiology of DED necessitates a more comprehensive therapeutic approach that can interfere with the intracellular signaling pathways, which are responsible for the development of pathological changes in the eyes of DED patients [6,7]. Therefore, a better understanding of the pathophysiological processes that regulate the development and progression of DED is crucially important for the establishment of new and more effective DED-related treatment approaches, which would significantly improve the quality of life of DED patients [1,6].

A large number of experimental and clinical studies have demonstrated that tear hyperosmolarity (THO) plays a crucially important role in the development and progression of DED [4,6,7]. Accordingly, osmoprotectants are frequently used to counteract the ocular surface damage caused by a hyperosmolar tear film [9,10,11,12]. Osmoprotectants act as “compatible solutes” that enter the cells, stabilize the cell membranes, maintain cellular hydration, and, due to their anti-oxidant and anti-inflammatory properties, protect the eye from oxidative stress and inflammation-induced injury [9,10,11]. L-carnitine, a substance found in osmoprotectants, has been shown to inhibit the activity of the transient receptor potential vanilloid 1 (TRPV1) ion channel in human corneal epithelial cells (CECs), suppress hypertonicity-induced increases in intracellular calcium ions (Ca^2+^), and prevent cell shrinkage [12,13]. Since increased and prolonged activity of the TRPV1 channel is associated with the aggravation of DED-related symptoms, topical administration of L-carnitine-containing osmoprotectants could have beneficial effects in DED treatment [12].

Although tear osmolarity testing is frequently used as a tool for DED diagnosis and treatment monitoring [5], the results of one recently conducted multi-center clinical study (“Dry Eye Assessment and Management (DREAM)”) showed only a weak correlation between tear osmolarity and DED-related symptoms [14]. In order to delineate the role of tear osmolarity in the pathogenesis of DED, in this review article, we summarized current knowledge related to the molecular mechanisms responsible for the development of tear hyperosmolarity (THO)-induced pathological changes in the eyes of DED patients and we briefly discussed the therapeutic potential of hypo-osmotic eye drops in DED treatment. In June 2023, a comprehensive literature review was undertaken using many databases (MEDLINE, EMBASE, and Google Scholar). The keywords used in our selection were: “dry eye disease”, “keratoconjunctivitis sicca”, “dysfunctional tear syndrome”, “tear hyperosmolarity”, “eye inflammation”, “immunomodulation”, and “therapy”. All journals were considered and the initial search retrieved 112 articles. The abstracts of all these articles were subsequently reviewed by two of the authors (CRH and VV) independently to check their relevance to the subject of this manuscript. Eligible studies had to delineate the molecular and cellular mechanisms responsible for the THO-related pathological changes in the eyes of DED patients, and their findings were analyzed in this review.

## 2. Molecular Mechanisms Responsible for THO Development

Tears are a lacrimal gland-sourced salty solution that lubricates and protects the corneal epithelium and inner surface of the eyelids from foreign particles and infectious agents [15]. THO is caused by alterations in tear composition and production in cases of poor aqueous tear flow, excessive tear evaporation, or a combination of these occurrences [16]. The composition of tears may vary based on their purpose. Basal tears contain water, electrolytes, and anti-bacterial proteins (lysozyme, lactoferrin, and lipocalin), which destruct bacterial cells walls or bind to iron, making it unavailable to the invading pathogens [17]. Emotional tears, on the other hand, contain higher levels of stress hormones and other chemicals, including leucine and enkephalin, which may reduce pain and improve mood. Additionally, emotional tears contain prolactin, which promotes the synthesis of immunoglobulin A (IgA) in plasma cells and enhances the anti-bacterial humoral immune response in the eyes [17]. Alterations in the concentration of electrolytes, proteins, and other solutes in the tears leads to the changes in tear osmolarity and represents one of the first indicators of lacrimal gland dysfunction [17].

## 3. The Impacts of Dysfunctional Neural Regulation of Lacrimal Gland Secretion on the Development of THO and DED Progression

Dysfunction in the neural control of glandular tear secretion plays a significant role in the development of THO and DED progression [18]. When sensory neurons in the cornea and conjunctiva detect changes in osmolarity, they transmit signals to the lacrimal glands, initiating tear secretion [18]. A large number of experimental and clinical studies have demonstrated that transient receptor potential (TRP) channels are mainly responsible for the neural regulation of tear production [18,19,20,21,22,23,24,25,26,27,28]. TRP channels belong to a heterogeneous superfamily of widely expressed, functionally varied cation channels that display intricate regulation patterns and are sensitive to a variety of stimuli, including THO [23,24]. Among various TRP channels, the transient receptor potential melastatin 8 (TRPM8) and transient receptor potential vanilloid 1 (TRPV1) are dominantly expressed in sensory neurons of the cornea and conjunctiva [22,23,24]. TRP channels are essential for normal neuronal functioning and play a crucial role in sensory perception, neuronal excitability, and synaptic transmission [25]. The activation of TRP channels in presynaptic terminals can modulate the release of neurotransmitters, affecting synaptic transmission and neuronal communication [25].

It should be emphasized that TRP channels are also expressed in non-neuronal corneal cells and in all three layers of the cornea [25]. These channels are essential for maintaining the health and functionality of the cornea, contributing to clear vision and overall ocular well-being. In addition to sensory transduction, TRP channels are involved in maintaining corneal integrity and promoting the repair and regeneration of injured tissue [25]. TRP channels affect the proliferation, differentiation, and migration of corneal epithelial and stromal cells, enabling remodeling of the extracellular matrix and wound healing [24,25]. Furthermore, TRP channels, particularly TRPM8 and TRPV4, are involved in regulating tear secretion and osmolarity. Activation of these TRP channels can stimulate tear production and maintain a stable tear film, preventing dry eye conditions [25].

In addition to sensory transduction, TRP channels play a crucial role in the regulation of corneal integrity, wound healing, and tear film homeostasis [22,23,24,25].

Although there are distinctly different functions of TRP channels between neuronal and non-neuronal cells, the activation mechanisms are the same [25]. After TRP channel activation, calcium release occurs through various pathways, depending on the specific TRP channel and cellular context [21,22,23,24,25]. Some TRP channels are involved in the regulation of store-operated calcium entry. In this process, TRP channels act as calcium-permeable channels located on the plasma membrane. When the endoplasmic reticulum (ER) calcium stores are depleted, usually due to receptor-mediated activation of phospholipase C (PLC) and subsequent inositol trisphosphate (IP3) production, the stromal interaction molecule (STIM) undergoes conformational changes and migrates to regions of the ER close to the plasma membrane, where it activates TRP channels [23,24,25]. This activation allows Ca^2+^ ions to enter the cytoplasm through the TRP channels, replenishing the depleted ER stores. Some other TRP channels can directly allow Ca^2+^ ions to enter the cell upon activation. These channels have a high permeability to calcium and are often referred to as calcium-permeable TRP channels. Activation of these TRP channels leads to conformational changes that open the channel pore, allowing Ca^2+^ ions to flow into the cytoplasm from the extracellular environment. The influx of calcium through these TRP channels can rapidly increase cytoplasmic calcium levels, triggering various cellular responses [23,24,25].

Following the evaporation of the tear film, activated TRPM8 receptors allow Ca^2+^ ions to enter the cell [20,21]. The increased intracellular calcium concentration activates calcium-dependent protein kinases (protein kinase C (PKC) and calcium/calmodulin-dependent protein kinase II (CaMKII)). PKC and CaMKII phosphorylate downstream target proteins, resulting in the activation of transcriptional factors (nuclear factors of activated T cells (NFAT) and cAMP response element-binding proteins (CREB)), which enhances the expression of genes that regulate the production and secretion of neuropeptides (substance P and calcitonin gene-related peptide (CGRP)) [20,21]. These neuropeptides stimulate tear production in lacrimal glands. Upon binding to neurokinin-1 and CGRP receptors, substance P and CGRP initiate the activation of cyclic adenosine monophosphate (cAMP) and PLC-driven intracellular signaling cascades within the lacrimal gland cells [20,21]. The cAMP pathway stimulates the opening of chloride (Cl-) channels, allowing the secretion of Cl- ions into the lacrimal gland ducts. This is followed by the efflux of sodium (Na+) ions and water, creating an osmotic gradient that drives the secretion of tears. Activated PLC catalyzes the hydrolysis of phosphatidylinositol 4,5-bisphosphate (PIP2) into inositol 1,4,5-trisphosphate (IP3) and diacylglycerol (DAG) [19,20]. IP3 diffuses into the ER of lacrimal gland cells and binds to IP3 receptors, causing the release of Ca^2+^ ions from intracellular stores. The increase in intracellular calcium concentration activates calcium-activated chloride channels (CaCCs) present on the apical membrane of lacrimal gland cells [20,21]. These channels allow the efflux of chloride ions (Cl-) from the cytoplasm into the lumen of the lacrimal gland ducts. The efflux of chloride ions creates an osmotic gradient, which drives the movement of sodium ions (Na+) and water from the interstitium into the lumen of the ducts through paracellular and transcellular pathways [19,20]. Along with fluid secretion, the activation of the PLC pathway also stimulates the secretion of electrolytes into the lacrimal gland ducts. The combined effect of fluid and electrolyte secretion in the lacrimal gland results in tear production [20,21]. Since TRPM8/neuropeptide-dependent activation of cAMP and PLC in lacrimal gland cells is essential for optimal tear production and proper hydration of the ocular surface, any dysfunction of the TRPM8 receptors leads to reduced tear production and causes dryness of the ocular surface [20,21,22,23].

In addition to its effect on tear secretion, TRPM8 modulates the expression of genes that regulate the syntheses of tight junction proteins (occludin, claudin), adhesion molecules (E-cadherin), and actin-binding proteins (myosin light chain), maintaining the stability and integrity of the epithelial barrier on the ocular surface [22,23]. Accordingly, dysfunction of the TRPM8 receptors causes an increased permeability of the ocular surface epithelium, enhanced evaporation of tears, decreased tear film stability, and dryness of the ocular surface [23,24]. Also, TRPM8 dysfunction causes heightened ocular surface sensitivity, discomfort, and pain, which are the main clinical symptoms reported by DED patients [22,23,24].

These findings are in line with results obtained by other researchers in two pilot clinical trials, which revealed that topical administration of the TRPM8 agonist (cryosim-3) significantly increased basal tear production and lessened neuropathic pain in DED patients, indicating that the use of TRPM8 agonists could be considered as a potential new therapeutic approach for the treatment of DED-related ocular discomfort [29,30].

Approximately half of corneal TRPM8+ neurons express the TRPV1 channel that allows the passage of calcium, sodium, and potassium ions [31]. TRPV1 is highly expressed in corneal and conjunctival TRPM8+ sensory neurons, and in anterior eye samples and trigeminal ganglia of mice and rats with DED [22]. An increased osmolarity of the tear film triggers a conformational change in these TRPV1 channels, leading to their opening. Massive influxes of cations cause disruption to cellular homeostasis [23]. Accordingly, THO-dependent prolonged activation of TRPV1 channels causes tear film instability and compromises the integrity of the epithelial barrier in the eyes, exacerbating DED [23,24]. Also, activation of TRPV1 in human CECs importantly contributes to the development of ocular inflammation in DED patients [32]. TRPV1-dependent increases in the intracellular concentration of calcium, which leads to the activation of mitogen-activated protein kinase (MAPK) and transcriptional factor NF-κB, resulting in an enhanced production of inflammatory cytokines (IL-6 and IL-8) [32]. Additionally, the nociception experienced with DED is a result of TRPV1 activation on the ophthalmic branch of the corneal trigeminal nerve endings [33]. Bearing in mind that a continuous activation of TRPV1 aggravates DED [22,23,24], Benitez-Del-Castillo and colleagues conducted phase I and II pilot clinical trials to test the safety and efficacy of SYL1001 (TRPV1-specific short interfering RNA) in DED patients [34]. Topical administration of SYL1001 significantly extended tear break-up time, prevented THO, improved the disease index score of ocular surfaces, and attenuated conjunctival hyperemia in the eyes of DED patients, without causing any severe side effects [34].

### The Impact of Hormones, Medication, and Environmental Factors on Tear Osmolarity

Hormones (cortisol, aldosterone, thyroid hormones, etc.), medications (antihistamines, beta-blockers, diuretics, isotretinoin, anti-cholinergics, etc.), and environmental factors all affect tear osmolarity and regulate tear-dependent eye protection. Cortisol can increase tear osmolarity by enhancing the expression of sodium channels in CECs, which can lead to an increase in the transportation of sodium ions into the tear film. Aldosterone, on the other hand, can increase the production of sodium–potassium pumps in the cornea, which can lead to an increase in the transportation of potassium ions out of the tear film [15,16,17]. This can also lead to an increase in tear osmolarity. Thyroid hormones (thyroxine (T4) and triiodothyronine (T3)) can affect the metabolism of cells in the lacrimal gland, which can influence the production and composition of tears, affecting tear osmolarity [35]. The increased production of thyroid hormones in overactive thyroid glands usually results in increased tear production and decreased tear osmolarity, as the metabolism of cells in the lacrimal gland is increased. Therefore, epiphora, watery eyes, is frequently observed in patients with hyperthyroidism. On the other hand, patients with hypothyroidism, or an underactive thyroid gland, may have a decreased tear production and an increased tear osmolarity, as the metabolism of cells in the lacrimal gland is decreased [16,17]. Lucius and colleagues showed that a thyroxine metabolite, 3-iodothyronamine (3T1AM), could act as a TRPM8 agonist. A significant increase in the intracellular Ca^2+^ influx was observed in 3T1AM-exposed immortalized human CECs, suggesting that 3T1AM-type analogs should be used as the leading compounds in developing drugs that would directly activate TRPM8 and reduce desiccation of the anterior ocular surface epithelia [35].

Since THO induces pathological changes in corneas, conjunctivas, and lacrimal glands, and elicits a detrimental immune response in the eye, THO is considered an important step in the development of DED [6,16].

## 4. THO-Related Pathological Changes in the Eyes of DED Patients

Lid margins, preocular tear film, and conjunctival sacs display distinct tear volumes and different tear osmolarities [36]. Decreased tear flow and environmental factors (low relative humidity, high wind speed, raised air temperature) may increase tear osmolarity [37]; it is at its the lowest upon waking, and rises very quickly during the day, reaching baseline levels within 40 min [37,38]. Tear osmolarities are usually measured using samples drawn from the lower tear meniscus in resting conditions [38]. The average tear osmolarity in resting conditions, measured in lower tear menisci of healthy eyes in resting conditions, is 302 ± 9.7 mOsm/L. The ocular surface, when exposed to tears with osmolarities ranging from 292.3 to 311.7 mOsm/L, is protected from any harmful effects of osmolarity [38]. The most sensitive baseline for distinguishing normal eyes from those with mild–moderate DED is a tear osmolarity of 308 mOsm/L (measured in the lower meniscus), and the most definite cut-off is 316 mOsm/L [38]. Tear osmolarities of up to 519 mOsm/L have been reported clinically [39], and the highest levels of osmolarity have been hypothesized to be at sites of tear film break-up [40].

The tear film is composed of three distinct (lipid, aqueous, and mucin) layers, which work together to provide lubrication, protect the ocular surface from infections, and maintain clear vision [41]. The lipid layer reduces tear evaporation, the aqueous layer provides moisture and nutrients, and the mucin layer ensures the proper adherence and distribution of tears. Any disruption or imbalance in these layers can lead to tear film instability, which importantly contributes to THO development [41]. Tears are drained via the nasolacrimal system and lost via evaporation. Enhanced tear evaporation disrupts the layered structure of the tear film and increases its osmolarity [41,42]. Specifically, tear osmolarity will double if evaporation reduces the aqueous layer of tear film to half of its initial thickness, provided that any tear solutes, including salts, are not lost from the aqueous layer (for example, via diffusion out of the region) [6].

In addition to increased tear osmolarity, enhanced evaporation reduces the tear film thickness, disrupting its normal structure and making it more susceptible to break-up [37,38]. Evaporation-driven tear film rupture is observed using fluorescence imaging as black circular spots, linear streaks, or irregular pools on a yellow-green fluorescence background [41]. The tear film is refreshed by blinking, which, in people with healthy eyes, occurs with a frequency of 10 to 30 times per minute [37,38]. When blinking is slowed, tear film break-ups appear within 15 to 40 s, in individuals with healthy eyes. However, tear film break-up regions (“black spots/streaks”) have been observed in the eyes of DED patients every 2–3 s [42,43]. Importantly, these “black spots/streaks” arose in areas where the aqueous layer of the tear film was entirely ruptured down to the underlying mucin/corneal interface, leaving the underlying epithelial cells completely unprotected [42,43]. Since osmolarity in the tear film break-up regions of DED patients can be dramatically high (between 800 and 900 mOsM) [44], THO is recognized as a major cause of ocular surface damage in DED patients. The most damaging THO-dependent pathological changes mainly develop in local salinity spots, which arise in the black spots of tear breakup [40,43].

Since the process of osmoregulation, which guard the ocular surface epithelia and maintain normal vision, is affected when the osmolarity of the tears is greater than that of the epithelial cells, THO is considered as an initial, important step in the development, progression, and aggravation of DED [6,9,16]. Hypertonicity induces activation of the TRPV1 signaling pathway in CECs, resulting in the activation of nuclear factor kappa B (NF-κB) [44,45]. By triggering NF-κB signaling, THO induces apoptotic cell death in CECs and disrupts the integrity of the corneal epithelial barrier, leading to increased permeability and susceptibility to infection [44,45].

**Figure 2:**
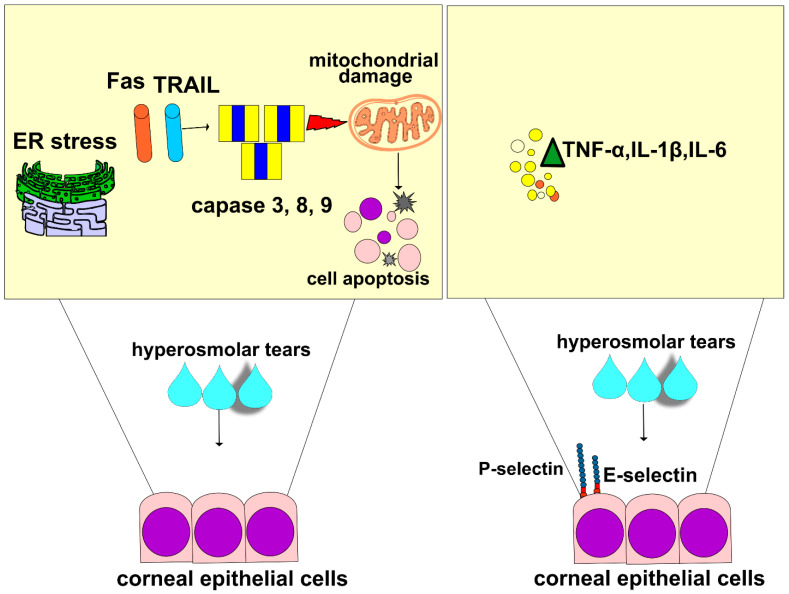
THO-induced changes in the phenotype and function of CECs. Apoptotic cell death is observed in THO-exposed CECs. THO induces stress in the ER and promotes mitochondrial damage by enhancing Fas- and TRAIL-dependent activation of caspase-3, capsase-8, and caspase-9, which results in apoptosis of THO-exposed CECs. Additionally, THO promotes an increased expression of E and P selectins and induces an enhanced production of inflammatory cytokines (TNF-α, IL-1β, IL-6) in CECs, which results in a massive influx of circulating immune cells in inflamed eyes of DED patients, resulting in the aggravation of ongoing inflammation.

In line with these findings are the results recently obtained by Guindolet and colleagues, who demonstrated that hyperosmotic environments caused the apoptosis of corneal and conjunctival epithelial cells by inducing dysfunction in the ER (Figure 2) [46]. The ER is cellular organelle that contains chaperones and folding enzymes, which facilitate the assembly of newly synthesized proteins, preventing their misfolding and aggregation [47]. A hyperosmotic microenvironment affects the transportation of secretory content in the ER and attenuates its capacity to fold proteins [46]. The resulting accumulation of unfolded proteins activates a cascade of stress-related signaling, referred to as the unfolded protein response (UPR), which transiently attenuates translation and increases protein folding in ERs of stressed epithelial cells [46]. An increased UPR activity, induced by THO, enhances the synthesis of transcription factor X-box binding protein 1 (XBP1), which directs the expression of chaperones and folding enzymes to restore homeostasis. If THO induces severe ER stress that cannot be attenuated by XBP1s, the UPR increases the activity of DNA damage-inducible transcript 3 protein (DDIT3), initiating apoptosis and the removal of stressed corneal and conjunctival epithelial cells. THO-dependent induction of ER-stress-associated programmed death of these cells results in the disruption of the ocular surface epithelial barrier, paving the way for the entrance of invading pathogens in the eyes of DED patients [46].

Additionally, THO-dependent activation of NF-κB in CECs is responsible for a massive influx of circulating leukocytes in inflamed eyes of DED patients [16,48]. THO-exposed CECs express high levels of adhesion molecules and massively produce pro-inflammatory cytokines (interleukin (IL)-1β, and tumor necrosis factor alpha (TNF-α)) which attract circulating immune cells to the inflamed corneas and lacrimal glands of DED patients. Upon binding to their receptors, IL-1β and TNF-α induce an increased expression of E and P selectins on the membrane of endothelial cells, enabling the adhesion, recruitment, and influx of circulating leukocytes in inflamed eyes [16,17]. Additionally, IL-1β and TNF-α stimulate the enhanced production of inflammatory cytokines in eye-infiltrated immune cells, creating a “positive inflammatory loop” that leads to the progression of DED [16,49]. IL-1β and TNF-α activate several kinases (mixed lineage kinase (MLK)-2/-3, transforming growth factor β-activated kinase 1 (TAK1), mitogen-activated protein kinase (MKK)-1, apoptosis signal-regulating kinase 1 (ASK1), MKK7, and Jun N-terminal kinase (JNK)) in eye-infiltrated dendritic cells (DCs) [16]. These kinases phosphorylate and activate transcriptional factors c-Jun and ATF2. Activated c-Jun and ATF2 translocate to the nucleus to induce an enhanced expression of pro-Th1 (IL-12) and pro-Th17 cytokines (IL-1β, IL-6, IL-23) in DCs [16]. Through the production of these inflammatory cytokines, DCs activate Th1- and Th17-specific transcriptional factors (T-box transcription factor TBX21 (T-bet), and RAR-related orphan receptor gamma (ROR-γ)) in naïve T cells, inducing their differentiation in effector, inflammatory, Th1, and Th17 lymphocytes. Th1 cell-derived interferon gamma (IFN-γ) and Th17 cell-sourced IL-17 and IL-22 activate macrophages and neutrophils in the inflamed eyes of DED patients, crucially contributing to the aggravation of DED [16,49].

## 5. THO-Dependent Activation of T Cell-Driven Detrimental Immune Responses in the Eyes of DED Patients

**Figure 3:**
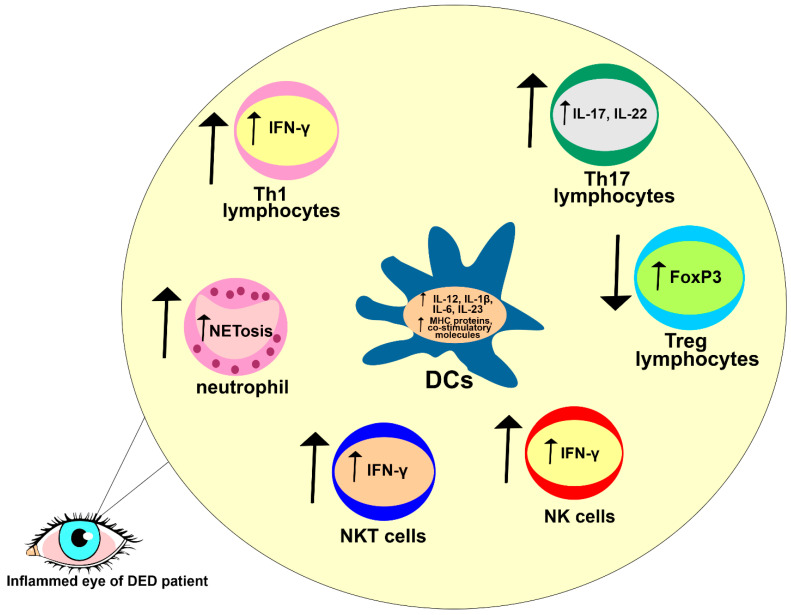
Detrimental immune response in the eyes of DED patients. Increased expressions of MHC proteins and co-stimulatory molecules were noticed in eye-infiltrated DCs of DED patients. DCs of DED patients have increased capacities for the production of inflammatory, pro-Th1 (IL-12), and pro-Th17 cytokines (IL-1β, IL-6, IL-12, IL-23), crucially contributing to the generation of detrimental Th1 and Th17 cell-driven immune responses. Accordingly, an increased number of IFN-γ-producing Th1, NK, and NKT cells and an increased number of IL-17- and IL-22-producing Th17 cells were observed in the eyes of DED patients. Additionally, THO promotes NETosis in eye-infiltrated neutrophils, importantly contributing to the progression of DED.

THO-dependent apoptosis of CECs disrupts the integrity of the mucosal barrier of the ocular surface, enabling a massive influx of invading pathogens [50]. Antigens that bypass the THO-injured epithelial barrier are captured by skillful antigen-presenting DCs that reside in the corneal epithelium. THO induces an increased activation and maturation of DCs and promotes their recruitment in inflamed corneas and conjunctivas by enhancing the expression of adhesion molecules on epithelial and endothelial cells [50,51]. Accordingly, one study observed a significantly increased number of activated DCs in the ocular surfaces of THO mice compared to healthy controls (Figure 3) [51].

Upon the engulfment of invading pathogens, activated DCs process their antigens and present them to naïve CD4+ T lymphocytes in regional lymph nodes. In one study, the DCs isolated from eye-draining lymph nodes of THO mice had better antigen-presenting properties than the DCs that infiltrated regional lymph nodes of control mice [16]. Higher expressions of molecules that are responsible for DC-dependent activation of naïve T cells (co-stimulatory and major histocompatibility complex (MHC) proteins) were observed on the membranes of THO-primed corneal and conjunctival DCs, indicating that a hyperosmolar microenvironment promotes efficient antigen presentation and the optimal activation of T cells [16,50]. Accordingly, a higher number of activated CD4+T cells were observed in the eye-draining lymph nodes of THO mice than in control animals [51].

THO promotes MKK7- and JNK-dependent activation of c-Jun and activating transcription factor 2 (ATF2) in corneal and conjunctival DCs. Upon the activation of c-Jun and ATF2, DCs enhance the production of inflammatory cytokines IL-12, IL-1β, IL-6, and IL-23 [50]. While DC-derived IL-12 triggers Th1-driven eye inflammation, DC-sourced IL-1β, IL-6, and IL-23 are responsible for the generation of Th17 cell-dependent injury in meibomian and lacrimal glands. Through the production of IL-12, DCs activate signal transducers and activators of (STAT)-1 and T-bet transcriptional factors in naïve CD4+ T lymphocytes, inducing their differentiation in effector IFN-producing Th1 cells. Similarly, by activating STAT-3 and RAR-related orphan receptor gamma T (RORγT) transcriptional factors in naïve CD4+T cells, DC-derived IL-1β, IL-6, and IL-23 induce the generation and expansion of inflammatory CD4+Th17 cells [50,51].

CD4+Th1 lymphocytes, in an IFN-γ dependent manner, and CD4+Th17 lymphocytes, in IL-17- and IL-22-depenent ways, induce the generation of inflammatory M1 and N1 phenotypes in the eye-infiltrating macrophages and neutrophils of DED patients [49]. These cells are valuable sources of inflammatory mediators, which contribute to the reduced expression of mucins, apoptotic death of corneal and conjunctival epithelial cells, and to the loss of goblet cells in inflamed eyes of DED patients [52]. Additionally, M1 macrophages and N1 neutrophils produce Th1 and Th17 cell-attracting chemokines (C-X-C motif ligand (CXCL)-9/-10, C-C motif ligand (CCL)-20), enabling massive influxes of CD4+ and CD8+ Th1 and Th17 cells in inflamed eyes of DED patients [52].

Th1 and Th17 lymphocytes, through the production of inflammatory cytokines (IFN-γ and IL-17), have been shown to induce disruption of the corneal epithelial barrier, trigger injury of the meibomian glands, and reduce tear production in the eyes of DED mice [52]. Also, antibody-dependent neutralization or genetic deletion of IFN-γ and IL-17 significantly attenuated eye inflammation and almost completely prevented corneal epithelial barrier dysfunction in the eyes of DED mice, confirming the crucially important pathogenic role of these inflammatory cytokines in the pathogenesis of DED. Th17 cell-derived IL-17 increases the expression of MMP-3 and MMP-9 which, in turn, induces disruption of the corneal epithelial barrier [53]. Th1 cell-sourced IFN-γ activates both extrinsic and intrinsic apoptotic pathways in CECs. The extrinsic or “death receptor” route is mediated by Fas ligand (FasL)- and TNF-related apoptosis-inducing ligand (TRAIL)-dependent activation of caspase-8, and the intrinsic or “mitochondrial” route is mediated by caspase-9. In further research findings, while exogenous administration of IFN-γ significantly increased caspase-3, -8, and -9-dependent apoptosis in conjunctival epithelial cells, pharmacological inhibition or genetic deletion of IFN-γ remarkably alleviated immune cell-driven injury of conjunctival epithelial and goblet cells in the eyes of DED mice [54]. Similar results have been observed in DED patients; IL-17-dependent corneal barrier disturbance was linked to an increased risk of corneal ulcers, corneal haze, and vision loss, while increased levels of IFN-γ was related to conjunctival epithelial squamous metaplasia, with progressive goblet cell loss [55].

Guzman and colleagues used passive transfer experiments to investigate whether detrimental Th1/Th17 cell-driven immune responses initiated by the disruptive effect of THO was sufficient to induce DED under mild desiccating stress [50]. Recipient mice were randomly divided in the two experimental groups to receive either T cells from the spleens and lymph nodes of THO mice or control (healthy) mice. Upon the passive transfer of T lymphocytes, recipient mice were exposed to mild desiccating stress (forced airflow without scopolamine). CD4+ and CD8+ T cells from THO mice homed more efficiently to lymph nodes than their control counterparts, and induced strong and potent detrimental immune responses that caused DED-related clinical symptoms in the recipient animals. A gradual reduction in tear production and damage and an increased irregularity of the corneal surface were observed in the eyes of the experimental animals that received the T cells from THO mice. These pathological changes were not observed in the animals that were injected with T cells from the control group, indicating the important role of THO in the priming of T cell-driven eye inflammation [50].

It has to be noted that the passive transfer of THO-primed T cells managed to induce DED in recipient animals only upon the depletion of immunosuppressive FoxP3-expressing CD4+ T regulatory cells (Tregs) [50]. Contrary to the inflammatory IFN-γ- and IL-17-producing Th1 and Th17 cells, Tregs produce immuno-modulatory factors (retinoic acid, IL-10, and transforming growth factor beta (TGF-β)), which attenuate the maturation and antigen-presenting properties of ocular DCs, prevent the generation of CD4+Th1 and Th17 lymphocytes in eye-draining lymph nodes, and inhibit the production of inflammatory cytokines (TNF-α and IL-1β) and Th1- and Th17-attracting chemokines (CXCL9, CXCL10, CCL-20) in N1 neutrophils and M1 macrophages, crucially contributing to the creation of anti-inflammatory microenvironments in the eyes of DED patients [56]. THO impairs immune homeostasis at the ocular surface by altering the ratio between inflammatory and regulatory T cells. THO favors the generation of pro-inflammatory (immunogenic), instead of immunosuppressive (tolerogenic), phenotypes in eye-infiltrated DCs [16,52]. While immunogenic DCs induce eye inflammation by promoting the activation of Th1 and Th17 cells, tolerogenic DCs create immunosuppressive microenvironments in the inflamed eyes of DED patients by inducing the expansion of Tregs [52]. A significantly reduced presence of immunosuppressive Tregs and increased numbers of activated CD69-expressing T lymphocytes, memory (CD62L^lo^ CD44^hi^) CD4+ T cells, and CD103-expressing CD8+ cytotoxic T cells have been observed in the draining lymph nodes of THO mice compared to healthy controls, indicating that THO-dependent modulation of DC phenotypes and functions altered the cellular make-up of eye-draining lymph nodes and promoted the progression of T cell-driven eye inflammation [50].

## 6. THO-Dependent Modulation of Eye-Infiltrated Neutrophils and Natural Killer Cells

Several lines of evidence have demonstrated that, in addition to macrophages, DCs, and T cells, THO modulates the phenotype and function of eye-infiltrated neutrophils and natural killer (NK) cells [16,52,57,58].

NETosis is a process in which neutrophils release their DNA and antimicrobial substances to trap and kill invading pathogens in the eyes of DED patients [52]. However, excessive NETosis can cause damage to the ocular surface and exacerbate ongoing inflammation, leading to further tissue damage and worsening of DED-related symptoms [52]. Tibrewal and colleagues showed that THO promoted NETosis in inflamed eyes, which leads to the aggravation of DED [56], and that neutrophils exposed to hyperosmolar conditions had morphological alterations (bigger and more rounded nuclei) and an increased capacity for NET production. To validate this influence of hyperosmolar circumstances on NET production, Tibrewal and colleagues restored iso-osmolar conditions, and, subsequently, NET formation was significantly reduced. Furthermore, the inclusion of NET formation inhibitors (such as staurosporine and anti-2 integrin) resulted in a reduction in the quantity of NETs under hyperosmolar conditions [56].

Because they are an early source of IFN-γ during the induction phase of experimental dry eye illnesses, natural killer (NK) and natural killer T (NKT) cells have been implicated in the immunopathogenesis of DED [58]. THO induces oxidative damage in CECs, which manifests with an increased expression of damage-associated molecular patters (DAMPs) and oxidative damage markers, and with increased productions of heme oxygenase-1 (HMOX1), cyclooxygenase-2 (COX2), and heat shock proteins (HSPs). NK and NKT cells recognize DAMPs and oxidative stress-related proteins that are expressed on the membranes of THO-exposed CECs [52,58]. This recognition can trigger the activation of NK and NKT cells, which results in a massive production of inflammatory cytokines, including IFN-γ and IL-17 [52]. NK and NKT cell-sourced IFN-γ enhances the expression of major histocompatibility complex (MHC) class II molecules and promotes the synthesis of IL-12 in DCs, enabling the generation of optimal Th1 cell-driven immune responses in the eyes of DED patients. Similarly, an increased production of the Th17 cell-attracting chemokine (CCL20) and enhanced synthesis of pro-Th17 cytokines (IL-1β, IL-6, and IL-23) were observed in NK and NKT cell-primed DCs [16,52]. The alleviation of THO, as well as the depletion of NK/NKT cells, significantly reduced the presence of activated DCs in the inflamed eyes of DED mice, alleviated their antigen-presenting properties, attenuated Th1 and Th17 cell-driven eye inflammation, down-regulated MMP-3 and MMP-9 expression, and reduced apoptosis of their corneal and conjunctival epithelial cells, confirming the important pathogenic role of THO in NK and NKT cell-driven inflammation in the eyes of DED patients [58].

## 7. Conclusions and Future Perspectives

**Figure 4:**
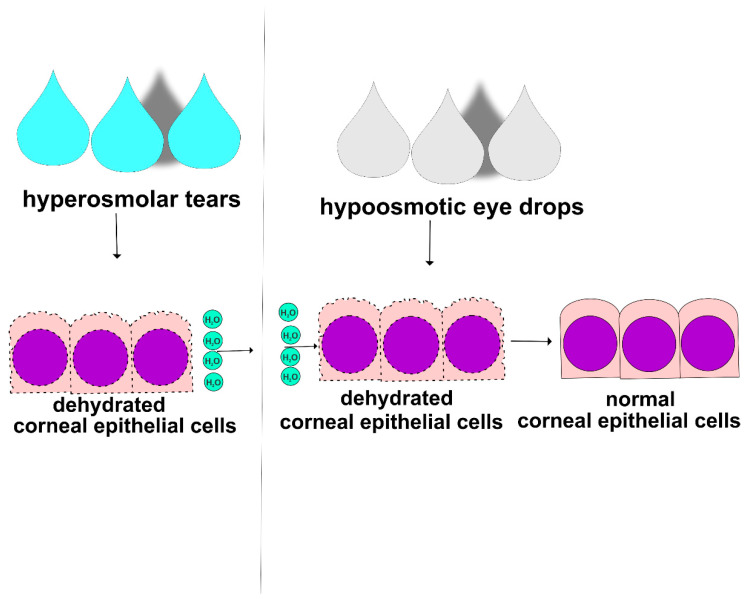
Therapeutic potential of hypo-osmotic eye drops in DED treatment. THO induces dehydration and consequent injury of CECs. Hypo-osmotic eye drops contain a lower concentration of salt and other solutes compared to the tears on the ocular surface. Accordingly, hypo-osmotic eye drops restore the natural osmotic balance of the ocular surface, improve tear film function, and attenuate THO-induced damage of the corneal epithelial barrier.

THO initiates and aggravates DED by: (i) inducing destruction of corneal and conjunctival epithelial barriers, (ii) enhancing Th1 and Th17 cell-driven immune responses, (iii) promoting NET formation, and (iv) stimulating NK and NKT cell-dependent inflammation in the eyes of DED patients [16,50,52,57,58]. Since THO plays an important pathogenic role in the development and progression of DED, hypo-osmotic eye drops can be used as a treatment for the alleviation of THO and DED [59]. Hypo-osmotic eye drops contain a lower concentration of salt and other solutes compared to the tears on the ocular surface, which can help to reduce THO and improve symptoms of dryness, burning, and irritation in the eyes of DED patients (Figure 4) [58]. When hypo-osmotic eye drops are instilled into the eye, they may help to restore the natural osmotic balance of the ocular surface, which can improve tear film function and decrease inflammation [59,60]. Hypo-osmotic eye drops may also decrease THO-induced damage of corneal and conjunctival epithelial barriers. However, it is important to note that hypo-osmotic eye drops may not be effective for all DED patients and that artificial tears, anti-inflammatory agents, or immunomodulatory drugs may be necessary to manage the underlying inflammation and improve their symptoms [60]. In line with these findings, it has to be noted that human CECs have the capacity to regulate their volume upon exposition to anisosmotic stress. Importantly, in the case of hypertonic stress, the time delay for the onset of regulatory volume increases was longer than that for regulatory volume decreases. Cell swelling developed within 30 s and then decreased over time [61]. Numata and colleagues showed that small interfering RNA (siRNA)-dependent silencing of TRPM7 significantly reduced the rate of cell volume recovery after osmotic swelling, indicating a crucially important role of TRPM7 in volume regulation of human epithelial cells [62]. Several lines of evidence have demonstrated that the expression of TRPV4 in human CECs was also important for maintaining the corneal epithelial barrier integrity during exposure to hypotonic stress [59,63,64]. When tear osmolarity decreases, the TRPV4 channels are activated, allowing the influx of Ca^2+^ ions into the cells. An increase in the intracellular concentration of calcium triggers a cascade of calcium-dependent signaling, which results in enhanced syntheses of substance P and CGRP. These neuropeptides subsequently stimulate the lacrimal gland to produce tears and restore tear osmolarity, preventing dryness-dependent damage to the ocular surface [63,64].

THO-induced pathological changes could also occur as a result of inadequate meibum consistency [16,65]. The meibum of patients with MGD-related DED has a toothpaste-like consistency and is not able to maintain stability and integrity of the tear film [65]. As a result, the tear film becomes more prone to evaporation, leading to an increased concentration of solutes in the remaining tears, which finally results in the development of THO-dependent pathological changes [16,65]. Accordingly, the use of intense pulsed light treatment and omega-3 fatty acids, phospholipids, triglycerides, and anti-oxidants (vitamin C and E)-enriched supplements, which manage to improve meibum consistency, stabilize the tear film, and attenuate THO-dependent injury and inflammation, should be considered as a supportive therapeutic approach for the treatment of DED patients [66,67].

Since THO induces apoptosis of corneal and conjunctival epithelial cells by triggering the activation of NF-κB signaling pathways in CECs [44,45,48], a combined use of NF-κB inhibitors and hypo-osmotic ophthalmic solutions could be used for the eradication of DED-related signs and symptoms in the injured and inflamed eyes of DED patients. Also, bearing in mind the important pathogenic role of NETosis, NK/NKT cells, and Th1/Th17 lymphocytes in the pathogenesis of THO-dependent eye inflammation [49,52], future experimental and clinical studies should investigate whether a combined administration of hypo-osmotic eye drops with immunosuppressive and anti-inflammatory drugs would have beneficial effects in the DED treatment.

Finally, it has to be noted that blinking and tear drainage almost immediately voids the artificial tears [68]. Therefore, the expectation that hypo-osmotic eye drops will manage to completely reduce DED-related symptoms must be considered with caution. Several strategies could be used to ensure longer-lasting effects of hypo-osmotic eye drops and prevent them from being voided by blinking and via tear drainage [69]. An addition of ointment-based supplements to hypo-osmotic eye drops could provide longer-lasting lubrication, reducing the risk of their rapid removal by blinking. Also, since preservatives in artificial tears sometimes cause irritation and increase the need for blinking, the use of preservative-free hypo-osmotic eye drops will reduce this risk and will provide longer-lasting effects. The use of punctual plugs, small devices which are inserted into the tear ducts to block tear drainage, could also provide hypo-osmotic eye drops with longer-lasting effects [69]. In line with all of these facts, although experimental and clinical studies have provided evidence of the crucially important pathogenic role of THO in the development and progression of DED, the therapeutic efficacy of hypo-osmotic eye drops should be confirmed in large randomized clinical trials before these drugs can be widely offered as new remedies for DED treatment.

## Data Availability

The data that are discussed in this article are presented in cited studies.
